# Impact of resident group action on orthopedic trauma care at a level 1 trauma center in Korea

**DOI:** 10.1097/MD.0000000000044818

**Published:** 2025-09-26

**Authors:** Kyung Wook Kim, Hee Gon Park, Hyun Woo Park, Ki Choul Kim, Sung Hyun Yoon, Dae Hee Lee, Young Kwang Shin, Su Hyeok Son, Bum-Jin Shim

**Affiliations:** a Department of Orthopaedic Surgery, Dankook University Hospital, Dankook University College of Medicine, Cheonan, Korea; b Department of Orthopedic Surgery, Yeungnam University Hospital, Yeungnam University College of Medicine, Daegu, Korea.

**Keywords:** epidemiology, medical issue, residents, trauma, traumatology

## Abstract

Residents in Korea have organized a movement against medical policies. People in the country are concerned that this can harm the medical system. We analyzed changes in trauma volumes and types among orthopedic patients and the mental status of doctors at a regional trauma center in Korea before and after residents took group action. We retrospectively reviewed the institutional trauma registry at our center from September 2023 to August 2024 to study changes before and after the group action of residents. We investigated demographic changes, including the overall number of patients and the mortality rate. We also divided the patients into groups according to their injury severity scores for subgroup analysis. Additionally, we investigated the patient health questionnaire-9 and Korean Epworth Sleepiness Scale (K-ESS) scores, which reflect the mental status of the remaining doctors, to assess their burden. The total number of patients decreased significantly from 404 in the first period to 150 in the second period (*P* < .001). The number of non-severe cases decreased from 361 to 104, while the number of severe cases remained the same (43 vs 46). In the severe group, other parameters, including the mortality rate, remained unchanged. Both the patient health questionnaire-9 and K-ESS scores significantly increased before and after the incident, indicating an increased burden on the remaining doctors. The orthopedic department experienced a decrease in non-severe case numbers and no significant changes in patient or mortality rates post-residential action, but an increased burden on the remaining doctors.

## 1. Introduction

Traumatic injuries are a major public health concern and can result in death.^[1]^ Korea has major regional trauma centers, each playing a vital role in its region.^[2]^ Trauma centers were established in each region so that trauma patients could reach them anywhere in the country within an hour.

In February of that year, group action by residents began in Korea as a movement against medical policy, and it has persisted without resolution to the present,^[3]^ resulting in the continued absence of residents in training. This policy has led to the exodus of many junior doctors, senior professors, and medical school students.^[4]^ Some professors resigned or departed indefinitely during protests against the government’s medical reform policies. This reform has caused great concern among the people of the country, and there are concerns that it could negatively impact the medical system.^[5]^

The trauma center system is closely related to reduced mortality and hospital stay, which have a direct impact on public health.^[6]^ Many university hospital systems have reported dramatic decreases in the number of patients who visit emergency rooms and trauma volumes.^[7]^ It is not unusual for ambulances to encounter delays while locating hospitals with the capacity to accommodate patients.^[8]^ In addition, the injury patterns of patients visiting emergency rooms have also changed.^[9]^ As a result, the current situation has become a major socioeconomic burden on the public healthcare system.

The current issue is crucial and could have major repercussions and changes in the Korean medical circle if the situation is prolonged.^[3,5]^ The actual medical situation we face is serious. The burden on the remaining doctors who filled the empty positions of the departed doctors became increasingly challenging over time. However, there is a paucity of literature regarding changes in trauma volume and distribution of injury patterns, as well as the burden on the remaining doctors. Our hospital, one of the largest regional trauma centers in Korea, is undergoing many changes, and we believe that it adequately represents the current medical situation. Therefore, the purpose of our study was to analyze the changes in overall trauma volumes and types of trauma in orthopedic trauma patients and to evaluate the mental health status of the remaining doctors at a single Level 1 regional trauma center before and after the group action of residents in Korea.

## 2. Materials and methods

### 2.1. Study design

This study’s design was approved by the institutional review board of the Dankook University College of Medicine/Dankook University Hospital (No. 2024-10-034), which waived the need for informed consent, owing to its retrospective nature. We retrospectively reviewed institutional trauma registries at our center. From September 2023 to August 2024, we divided the period into 6-month intervals to study the changes before and after the group action of residents. The first period was from September 2023 to February 2024, and the second period was from March 2024 to August 2024.

### 2.2. Measurements and outcomes

The following registry data were collected for patient demographics: age, sex, length of hospital stay, time to surgery, number of intensive care units (ICU), number of discharge/transfers, mortality rate, and severity of trauma according to the injury severity score (ISS), which is most widely used to define severely injured patients.^[10]^ Additionally, the number of patients admitted or discharged to the orthopedic department and the type of surgery according to the subspecialty were analyzed. Severely injured patients were defined as having an ISS ≥ 15, and non-severe patients were described as having an ISS < 15. We also evaluated the mental health status of all 8 remaining orthopedic physicians, including the severity of depression, stress, and daytime sleepiness, before and after the group action of residents, using the Patient Health Questionnaire (PHQ-9) and the Korean Epworth Sleepiness Scale (K-ESS).^[11,12]^

### 2.3. Statistical analysis

For subgroup analysis, differences were analyzed using an independent samples t-test for age, length of hospital stay, time to surgery, chi-square test for sex, the proportion of severely injured patients, proportion of patients who received ICU care, mortality rate, and type of surgery. In addition, for the mental health status of the remaining 8 doctors, the Wilcoxon signed-rank test was used to compare the patient health questionnaire-9 and K-ESS scores before and after the actions of the resident group. Statistical significance was set at *P <* .05. Statistical analyses were conducted using the IBM SPSS ver. 26.0 (IBM Corp., Armonk).

## 3. Results

### 3.1. Patient demographics

**Figure 1.**
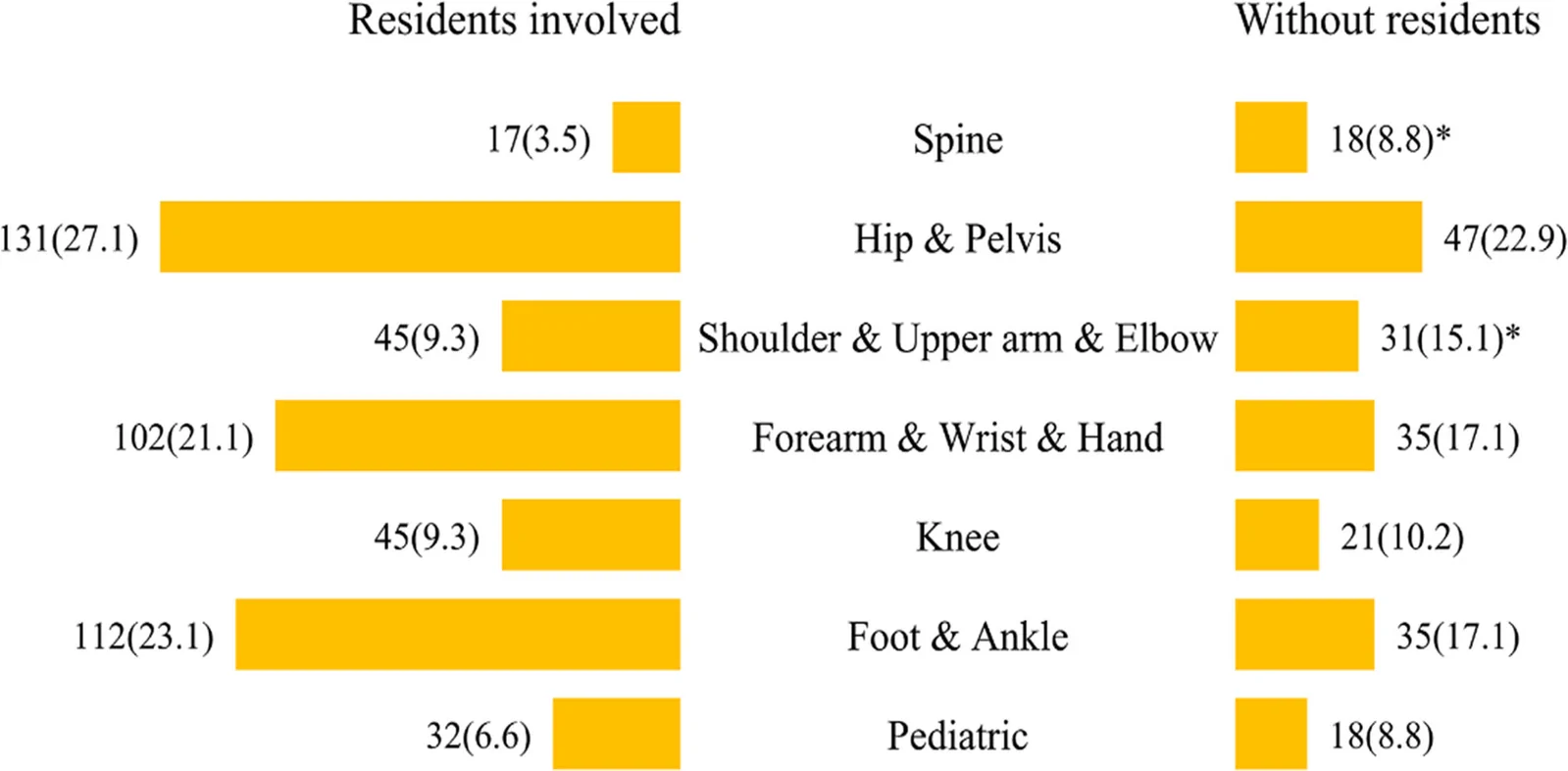
The number and percentage of each type of surgery before and after the group action of residents. The graph above depicts the change in the number and percentage of each type of surgery before and after the group action of residents. When comparing the 2 periods, there was a statistically significant increase in the percentage of spine and shoulder/upper arm/elbow surgery (*P* = .004 and *P* = .026, respectively). * Statistically significant difference. Numbers next to the bar graph represent the number(percentage) of each surgery type.

Table 1 shows the changes in demographics and other patient parameters. The total number of patients decreased significantly, from 404 in the first period to 150 in the second period (*P <* .001). When classified by ISS, the number of non-severe patients decreased from 361 to 104 between the 2 periods, whereas the number of severe patients remained similar (43 vs 46). The mean time to surgery and the number of ICU visits increased significantly (*P = *.025 and *P < *.001, respectively). However, hospital days and the number of discharges/transfers or mortality rates did not change significantly. Furthermore, when comparing the number (percentage) of each surgery type, the percentage of spine and shoulder/upper arm/elbow surgeries significantly increased (*P* = .004 and *P* = .026, respectively) between the 2 periods (Fig. 1).

**Table 1:** Changes in patient characteristics and injury sites based on the presence of residents.

Variable	Total (n = 554)	Resident involved (n = 404)	Without residents (n = 150)	χ^2^	*P*–value
Patient severity					
Non-severe	465 (83.9)	361 (89.4)	104 (69.3)	32.524	<.001*
Severe	89 (16.1)	43 (10.6)	46 (30.7)		
Age (year)	53.96 ± 24.57	55.03 ± 24.37	51.08 ± 24.94	–	.097
Sex					
Male	309 (55.8)	209 (51.7)	100 (66.7)	9.89	.002*
Female	245 (44.2)	195 (48.3)	50 (33.3)		
Hospital day (d)	21.17 ± 20.59	20.68 ± 20.67	22.49 ± 20.39	–	.354
Time to surgery (h)	73.07 ± 106.64	65.51 ± 89.52	93.34 ± 141.13	–	.025*
ICU care					
No	398 (71.8)	316 (78.2)	82 (54.7)	29.992	<.001*
Yes	156 (28.2)	88 (21.8)	68 (45.3)		
Outcome					
Discharge or transfer	550 (99.3)	401 (99.3)	149 (99.3)	0.009	.925
Death	4 (0.7)	3 (0.7)	1 (0.7)		
Type of surgery					
Spine	35 (5.1)	17 (3.5)	18 (8.8)	8.289	.004*
Hip, pelvis	178 (25.8)	131 (27.1)	47 (22.9)	1.288	.256
Shoulder, upper arm, elbow	76 (11.0)	45 (9.3)	31 (15.1)	4.978	.026*
Forearm, wrist, hand	137 (19.9)	102 (21.1)	35 (17.1)	1.447	.229
Knee	66 (9.6)	45 (9.3)	21 (10.2)	0.149	.700
Foot, ankle	147 (21.3)	112 (23.1)	35 (17.1)	3.159	.076
Paediatric	50 (7.3)	32 (6.6)	18 (8.8)	1.007	.316
Total	689 (100)	484 (100)	205 (100)	–	–

Values are presented as number (%) or mean ± standard deviation.

Statistical significance was set at *P* < .05, using independent samples *t*-test for age, hospital day, time to surgery, chi-squared test for sex, intensive care unit (ICU) care, outcome, and type of surgery.

* Statistical significance.

### 3.2. Subgroup analysis divided by injury severity scores

In the non-severe patients group, the number of patients receiving ICU care increased significantly between the 2 periods (Table 2). No significant changes were observed in any other parameters. Spine and pediatric surgery significantly increased from 2.8% to 8% and 6.9% to 12.4%, respectively (*P = *.008 and *P = *.040, respectively). However, no significant changes were observed.

**Table 2:** Comparison of patient characteristics and injury sites between non-severe and severe groups.

Variable	Non-severe (n = 465)					Severe (n = 89)				
	Total (n = 465)	Resident involved (n = 361)	Without residents (n = 104)	χ2	*P*-value	Total (n = 89)	Resident involved (n = 43)	Without residents (n = 46)	χ^2^	*P*-value
Age (year)	54.78 ± 25.14	55.38 ± 24.61	52.69 ± 26.92		.337	49.67 ± 20.93	52.07 ± 22.29	47.43 ± 19.55		.299
Sex										
Male	243 (52.3)	180 (49.9)	63 (60.6)	3.716	.054	66 (74.2)	29 (67.4)	37 (80.4)	1.958	.162
Female	222 (47.7)	181 (50.1)	41 (39.4)			23 (25.8)	14 (32.6)	9 (19.6)		
Hospital day (d)	18.97 ± 18.90	18.28 ± 17.65	21.38 ± 22.65	–	.140	32.64 ± 24.96	40.81 ± 31.08	25.00 ± 13.90	–	.003*
Time to surgery (h)	65.34 ± 74.34	61.90 ± 64.68	77.30 ± 100.44	–	.142	112 ± 200.53	96.73 ± 201.30	129.60 ± 202.28	–	.447
ICU care										
No	381 (81.9)	307 (85)	74 (71.2)	10.521	.001*	17 (19.1)	9 (20.9)	8 (17.4)	0.180	.671
Yes	85 (18.1)	54 (15)	30 (28.8)			72 (80.9)	34 (79.1)	38 (42.7)		
Outcome										
Discharge or transfer	461 (99.1)	358	103	0.016	.899	89 (100)	43 (100)	46 (100)	–	–
Death	4 (0.8)	3 (0.8)	1 (0.9)			0	0	0		
Type of surgery										
Spine	23 (4.1)	12 (2.8)	11 (8.0)	7.084	.008*	12 (9.3)	5 (8.2)	7 (10.3)	0.168	.682
Hip, pelvis	149 (26.6)	118 (27.9)	31 (22.6)	1.471	.225	29 (22.5)	13 (21.3)	16 (23.5)	0.091	.763
Shoulder, upper arm, elbow	53 (9.5)	38 (9.0)	15 (10.9)	0.467	.495	23 (17.8)	7 (11.5)	16 (23.5)	3.189	.074
Forearm, wrist, hand	112 (20.0)	90 (21.3)	22 (16.1)	1.761	.184	25 (19.4)	12 (19.7)	13 (19.1)	0.006	.937
Knee	52 (9.3)	39 (9.2)	13 (9.5)	0.009	.925	14 (10.9)	6 (9.8)	8 (11.8)	0.124	.725
Foot, ankle	125 (22.3)	97 (22.9)	28 (20.4)	0.371	.542	22 (17.1)	15 (24.6)	7 (10.3)	4.646	.031*
Paediatric	46 (8.2)	29 (6.9)	17 (12.4)	4.232	.040*	4 (3.1)	3 (4.9)	1 (1.5)	1.272	.259
Total	560 (100)	423 (100)	137 (100)	–	–	129 (100)	61 (100)	68 (100)	–	–

Values are presented as mean ± standard deviation or number (%).

Statistical significance was set as *P* < .05, using independent samples *t*-test for age, hospital day and time to surgery, chi-squared test for sex, intensive care unit (ICU) care, outcome and type of surgery.

* Statistical significance.

In the severe patients group, hospital stay showed significant decreases between the 2 periods, from 40.81 ± 31.07 to 25.00 ± 13.89 (*P = *.003). However, there were no significant changes in other parameters, including mortality rate. Only foot/ankle surgery showed a significant decrease from 24.26 to 10.3% between the 2 periods (*P = *.031).

### 3.3. The mental health status of the remaining doctors

Table 3 shows the mental health status of the doctors before and after the current issue. When comparing scores before and after the incident, both the patient health questionnaire-9 and K-ESS scores showed a significant increase, indicating that the burden on the remaining doctors increased.

**Table 3:** Comparison of PHQ-9 and K-ESS scores based on the presence of residents.

	Resident involved (n = 8)	Without residents (n = 8)	*P* value
PHQ-9 score	3.75 ± 0.71	4.63 ± 0.52	.020*
K-ESS score	4.87 ± 0.84	6.00 ± 0.76	.007*

K-ESS = Korean Epworth Sleepiness Scale, PHQ-9 = Patient Health Questionnaire.

Values are presented as mean ± standard deviation. Statistical significance was set at *P* <.05 using the Wilcoxon signed-rank test.

* Statistical significance.

## 4. Discussion

In the current study, the overall number of non-severe patients decreased after the group action of residents compared to the period before the group action of residents. However, no significant changes were observed in the number of severe patients. Furthermore, for severe patients, there were no differences in the mortality rate, and when examined by the orthopedic subspecialty, each part showed consistent treatment performance. One of the unique roles of trauma centers is the management of severely injured patients. However, the mental state of the remaining doctors was poor, suggesting that the burden on university hospital professors increased.

Several factors, including population, industry, and economic level, affect the epidemiology of trauma in a region.^[13]^ Among these, the influence of medical policies and the performance of medical personnel is essential.^[14]^ Korea is currently facing a medical crisis that has significantly impacted public healthcare systems, particularly regional emergency trauma centers.^[3–5]^ Recent strikes by medical professionals, including residents, have led to disruptions in the healthcare system with serious consequences. One notable issue is the increase in mortality rate, especially among non-severe patients in regional emergency rooms. Between January and July 2024, the mortality rate of non-severe patients increased by 39.6% in local emergency rooms compared to last year.^[15]^ This alarming increase contrasts with the decrease in mortality rates for severely injured patients at larger regional trauma centers, where the mortality rate decreased by 47.5%.^[15]^ In the current study, the number of non-severe cases decreased in our registry, and there were no significant changes in the mortality rate. This finding implies that non-severe patients may not have been admitted to the emergency room and may have been transferred to nearby hospitals that can manage them.^[16]^ Systematically, linkages with lower-level hospitals in the community are well established in regional trauma centers. However, our study did not provide information on how the patients were treated after their transfer to other hospitals. Unexpected events may have occurred during transport, and we cannot be sure of changes in the emergency patient’s condition. Therefore, it is difficult to conclude that the emergency system was well-maintained based on the results of this study. We need to be more alert, analyze the situation more comprehensively, and observe this issue more seriously.

The ongoing shortage of medical personnel has led to increased strain on doctors and paramedics, and some emergency centers have limited their medical services to certain conditions, such as dermatology and ophthalmology.^[17]^ Consequently, there are growing concerns about the impact of the current issue on vulnerable patients, particularly in smaller medical centers, where medical personnel are even more limited. In the current study, there was no significant change in the overall number of hip/pelvis and pediatric patients, which were departments with fewer orthopedic patients. In addition, the number of spine, shoulder, upper arm, and elbow surgery has increased. Otherwise, there were no significant changes, and it was confirmed that the overall orthopedic surgery was well-maintained. As it is challenging to perform spine and upper extremity surgery at a nearby orthopedic specialist hospital, the proportion of initially visiting patients is thought to have increased. Although the overall number of surgical procedures has decreased, the relative number of spine and upper extremity patients who initially visited the hospital is thought to have increased because it is challenging to perform spine and upper limb surgery at nearby specialized hospitals. Regarding overall performance, there were no significant changes in mortality rates or length of hospital stay between the 2 periods. Therefore, it can be inferred that they were well-managed. However, this study did not reveal how non-severe cases were managed and involved a relatively small number of patients. Therefore, caution should be exercised when interpreting the results.

Due to medical personnel shortages, most university hospitals have been forced to reduce outpatient department services, surgery, and emergency room care.^[17]^ Although the number of non-severe patients visiting hospitals has decreased since the implementation of the government policy to increase payments for emergency care services, the number of severe patients has not changed significantly. As a regional trauma center, our hospital performed reliably during a medical crisis. Therefore, the main role of trauma centers was fulfilled. However, these results imply the hidden efforts of those who remain in the absence of trainee doctors. This effort has been maintained to date. However, what will happen in the future cannot be guaranteed. This is because the accumulated work time increased. Even now, junior professors quit because of poor working conditions coupled with fatigue.^[18]^ The burden on understaffed and underresourced emergency rooms is mounting, and we are afraid that this situation may become unsustainable. It has been reported that the burden on medical personnel, including the remaining doctors, is increasing, and the resignation of professors is accelerating. Therefore, fundamental roles of professors, such as research and education, are likely to be diminished as well. Ultimately, this could pose a serious threat to the medical system that is directly related to public health. Therefore, vigilant nationwide research is required.

This study has several limitations. First, it was a retrospective cohort study conducted at a single trauma center. Hence, it may be difficult to generalize the results, and it can affect the patient population through several factors, such as the season or socioeconomic status of the region. Second, the mortality rate of transferred non-severe patients could not be analyzed because this study only analyzed the data from our institution. Finally, although there was a deterioration in the mental status of the remaining doctors, we could not analyze and quantify the workload or stress experienced by medical professionals, such as working hours or the number of on-call visits. Additionally, this study included all 8 orthopedic professors remaining in our institution; however, the sample size was small and limited to a single institution. Therefore, the findings may not be generalizable to all physicians or institutions.

Nonetheless, to our knowledge, this is the first report of a single Level 1 regional trauma center with a detailed analysis of the current changes in patients following medical issues in Korea, using the ISS to divide the patient severity group. Therefore, this study provides valuable information for understanding the current situation. Further nationwide, multicentre statistics and studies are required to understand the current situation comprehensively.

## 5. Conclusions

In the orthopedic department, the overall number of non-severe patients significantly decreased after the group action of residents compared with the period before the group action of residents. Additionally, there were no significant changes in the number of severe patients or mortality rate. However, the burden on the remaining doctors increased significantly.

## Author contributions

**Conceptualization:** Kyung Wook Kim, Hee Gon Park, Ki Choul Kim, Young Kwang Shin, Bum-Jin Shim.

**Data curation:** Hyun Woo Park, Ki Choul Kim, Bum-Jin Shim.

**Formal analysis:** Sung Hyun Yoon, Dae Hee Lee, Bum-Jin Shim.

**Funding acquisition:** Kyung Wook Kim, Bum-Jin Shim.

**Investigation:** Hee Gon Park, Su Hyeok Son.

**Methodology:** Hee Gon Park, Su Hyeok Son.

**Project administration:** Kyung Wook Kim, Bum-Jin Shim.

**Resources:** Sung Hyun Yoon, Dae Hee Lee.

**Software:** Ki Choul Kim, Sung Hyun Yoon, Dae Hee Lee.

**Supervision:** Kyung Wook Kim, Bum-Jin Shim.

**Validation:** Hee Gon Park, Ki Choul Kim.

**Visualization:** Sung Hyun Yoon, Dae Hee Lee.

**Writing – original draft:** Kyung Wook Kim, Bum-Jin Shim.

**Writing – review & editing:** Kyung Wook Kim, Bum-Jin Shim.
